# Untensed fibronectin fibers: a novel hallmark of microthrombi

**DOI:** 10.3389/fcvm.2025.1627917

**Published:** 2025-08-08

**Authors:** Arnaud Miéville, Petra Wolint, Nikola Cesarovic, Volkmar Falk, Viola Vogel

**Affiliations:** ^1^ Laboratory of Applied Mechanobiology, Department of Health Sciences and Technology, Institute of Translational Medicine, ETH Zürich, Zürich, Switzerland; ^2^ Department of Health Sciences and Technology, ETH Zürich, Zürich, Switzerland; ^3^ Department of Cardiothoracic and Vascular Surgery, Deutsches Herzzentrum der Charite (DHZC), Berlin, Germany

**Keywords:** MINOCA, mechanobiology, fibronectin, myocardial infarction, extracellular matrix (ECM)

## Abstract

**Background:**

Myocardial Infarction with Non-Obstructive Coronary Arteries (MINOCA) accounts for up to 15% of acute Myocardial Infarction (MI) cases and presents significant diagnostic and therapeutic challenges. The specific targeting of microthrombi involved in microthrombi-induced MINOCA with molecule-specific precision has been challenging due to their omnipresence in the bloodstream, highlighting the need for novel biomarkers and imaging strategies. Fibronectin, one of these omnipresent extracellular matrix (ECM) proteins, exists in distinct physical states in healthy versus diseased tissues, presenting stretched versus untensed fibers, which may serve as potential diagnostic and therapeutic targets.

**Methods:**

The peptide FnBPA5, a highly specific probe that binds selectively to untensed fibronectin fibers, as its multivalent binding motif is destroyed upon fiber stretching, was employed here to assess fibronectin's fiber tension in microthrombi before and after the onset of MINOCA in a pig model of autologous microthrombi-induced MINOCA.

**Results:**

Loss of fibronectin fiber tension was identified here as a novel key feature of microthrombi in a pig model of autologous microthrombi-induced MINOCA, whereas fibronectin fibers in the surrounding healthy myocardium remained highly stretched. FnBPA5 can thus effectively visualizes fibronectin's physical signature, thereby distinguishing microthrombi from the surrounding healthy tissue.

**Conclusion:**

These findings underscore FnBPA5's unique capacity to discriminate not merely the presence of an abundant ECM molecule within a thrombus, but its distinct physical conformation. FnBPA5 enables the selective detection of microthrombi in coronary arteries by targeting untensed fibronectin fibers, a novel mechanical biomarker of microthrombi. Targeting a protein's physical state with high specificity makes FnBPA5 a promising tool for advanced microthrombi detection and for mechano-therapeutic strategies involving the targeted delivery of therapeutic agents.

## Introduction

Cardiovascular disease (CVD) remains the leading cause of morbidity and mortality worldwide, accounting for an estimated 17.9 million deaths annually (1). Among its various manifestations, acute myocardial infarction (MI) is a major contributor to CVD-related mortality and disability. Acute MI has traditionally been associated with obstructive coronary artery disease (CAD), resulting in ischemic injury, subsequent myocardial necrosis and heart failure (2, 3). However, a subset of patients presents with a similar clinical presentation to MI despite the absence of significant coronary artery obstruction on angiography. This condition, known as Myocardial Infarction with Non-Obstructive Coronary Arteries (MINOCA), is increasingly recognized as a distinct clinical entity. Defined as ischemic myocardial damage without angiographic evidence of ≥50% coronary stenosis, MINOCA accounts for up to 15% of all acute MI cases (3–6). Unlike traditional MI caused by obstructive atherosclerosis, MINOCA is a heterogeneous condition that can result from a variety of pathophysiological processes, including coronary atherosclerosis, vasospasm, thromboembolism, and spontaneous coronary artery dissection. MINOCA is therefore considered a working diagnosis, encompassing multiple potential underlying mechanisms which, despite its relatively high prevalence, make its diagnosis and management particularly challenging (4–6). Understanding the pathophysiology, diagnostic challenges, and management strategies of MINOCA is therefore crucial for optimizing patient outcomes.

To advance our knowledge, Cesarovic et al. developed a translational microthrombi-induced MINOCA pig model to specifically study this subtype of MINOCA in depth, with the aim of advancing the development of new therapeutic and diagnostic strategies (4). In this model, they produce autologous microthrombi, which are then injected into one of the main epicardial arteries to induce the onset of microthrombi-induced MINOCA. Microthrombi are small (micron size), fibrin- and platelet-rich thrombotic aggregates that form in the microvasculature or occur as microemboli and can obstruct blood flow without causing significant coronary artery stenosis but still contribute to myocardial ischemia and injury by impairing perfusion at the microvascular level (4, 7–9). The use of tools that specifically target microthrombi in coronary arteries, in combination with advanced imaging modalities or treatments, could be highly beneficial for the development of both new diagnostic and therapeutic approaches, leading to improved management of microthrombi-induced MINOCA. Fibronectin is a ubiquitous glycoprotein that plays a vital role in tissue repair and the wound healing process. Plasma fibronectin circulates in the blood and is crucial for blood coagulation, clot formation, and the development of microthrombi by being incorporated into the fibrin clot, contributing to platelet function, fibrin network stabilization, and mediating hemostasis (10). Even in mice lacking both of the best-known platelet ligands, von Willebrand factor and fibrinogen, they still form occlusive thrombi in injured arterioles as their platelets accumulate excessive amounts of fibronectin (11). The major involvement of Plasma fibronectin in microthrombi formation could be used as potential biomarker for detecting microthrombi-related cardiovascular events.

Since most molecules targeted in thrombi are ubiquitous and not restricted to circulating microthrombi, we asked whether the physical state of certain proteins, i.e., stretched vs. untensed, could potentially be exploited. Since knowledge about the tension of any type of ECM fiber in blood clots is very limited, we investigated here whether fibronectin fibers are stretched or untensed in blood clots circulating through the vasculature. While fibronectin fibers are stretched in healthy organs (12, 13), the presence of untensed fibronectin fibers in the extracellular matrix (ECM) in pathologically remodeled ECM has emerged as a hallmark of various inflammatory conditions (14, 15), as well as cancer (12, 13, 16, 17) and fibrotic diseases (18). These discoveries were enabled by the systematic development and validation of a mechano-regulated tension sensor in recent years (12, 19, 20). The peptide FnBPA5, which specifically binds to untensed fibronectin fibers with nM affinity, recognizes fibronectin's N-terminal domains FnI_2–5_ with high specificity if fibronectin is in equilibrium. However, the peptide's binding affinity is mechanically downregulated upon fiber stretching, as this disrupts its multivalent binding motif (19–21). Fibronectin fiber stretching assays confirmed the high affinity of FnBPA5 for untensed fibronectin fibers, while increasing fiber strain progressively reduced its binding (12). This was further validated by labeling native fibronectin fibers in cell culture using two complementary strain probes, a fibronectin-fluorescence resonance energy transfer (FRET) probe (22, 23) and FnBPA5, which showed a strong correlation between the two readouts across a broad range of fibronectin fiber strains (12, 24). In contrast, staining with a polyclonal fibronectin antibody enables the detection of all fibronectin fibers, regardless of their physical state or strain (12, 13, 19, 20). Not only can this peptide be used to assess the presence of untensed fibronectin fibers, but it also holds great potential for both imaging and targeted therapy (12, 18). By linking FnBPA5 to fluorophores, radioligands, or therapeutic agents, it can specifically target tissues containing untensed fibronectin fibers (25), such as in cancer and fibrotic stroma in mice (12–15) and human (17, 18).

In this study, we evaluated the presence of untensed fibronectin fibers in the stroma of microthrombi before and after the onset of microthrombi-induced MINOCA to determine whether FnBPA5 could serve as a potential tool for microthrombi detection or MINOCA treatment. Our data show that microthrombi are highly enriched in untensed fibronectin fibers and can be detected in cardiac muscle using our peptide sensor, suggesting that our probes, whether linked to imaging modalities or therapeutic agents, could aid in the management of microthrombi-induced MINOCA.

## Results

Microthrombi were obtained from the Center for Preclinical Development, University Hospital Zurich. They were generated by performing multiple carotid artery crushes using a surgical clamp on female pigs (82 ± 5 kg; 4–5 months), as described in the study by Cesarovic et al. (4). 60 minutes after artery crushing, microthrombi were harvested, flash-frozen in liquid nitrogen, and stored at −80°C until cryosectioning and immunofluorescence (IF) staining.

### Pig microthrombi harvested after artery crushing show enhanced relaxation of fibronectin fibers

**Figure 1:**
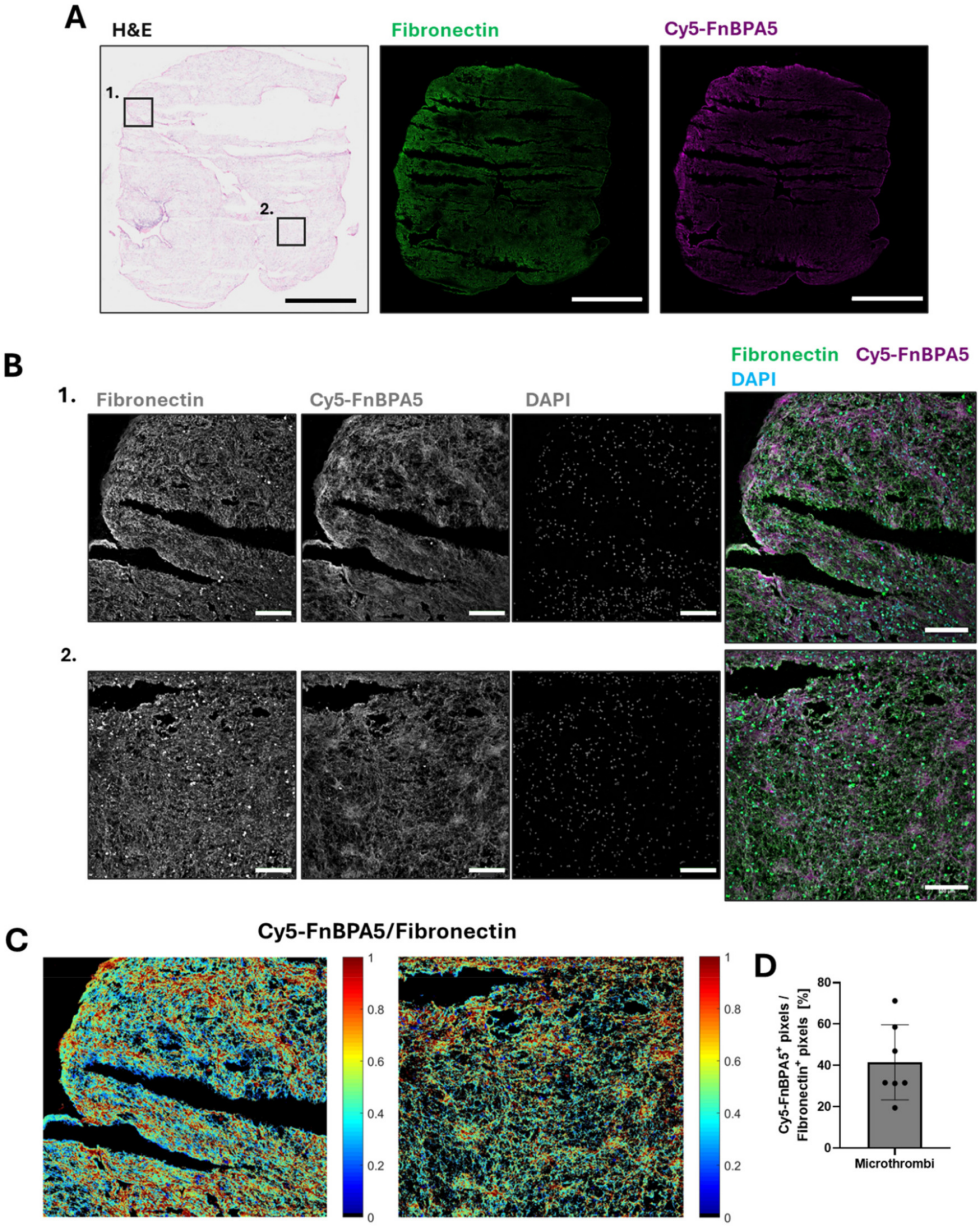
Porcine microthrombi are enriched in untensed fibronectin fibers. **(A)** Representative H&E stains and confocal images of cryosections of porcine microthrombi prior injection into myocardial arteries. Sections were stained with a polyclonal fibronectin antibody (green) to visualize the presence of all fibronectin fibers, co-stained with Cy5-FnBPA5 tension probe (magenta) to visualize the untensed fibronectin fiber pixels. Scale bar: 1,000 µm. **(B)** Higher resolution zoom-in confocal images stained with a polyclonal fibronectin antibody (green) to visualize the presence of all fibronectin fibers, co-stained with the Cy5-FnBPA5 tension probe (magenta) to visualize the untensed fibronectin fiber pixels and DAPI to visualize the cell nuclei. Scale bars of zoom-in images: 100 µm. **(C)** The Cy5-FnBPA5/fibronectin ratio shows the tensional heterogeneity within the ECM at the tissue scale. **(D)** Pixel density for the Cy5-FnBPA5 signal was assessed as the percentage of positive pixels above a defined threshold for Cy5-FnBPA5, normalized to the total number of positive pixels above a defined threshold for the fibronectin polyclonal antibody.

Stitched overview images of H&E stains for a representative microthrombi prior injection into the myocardial arteries, along with corresponding IF confocal images stained with a polyclonal antibody to visualize all fibronectin fibers and Cy5-FnBPA5 to visualize untensed fibronectin fibers from adjacent cryosections, are shown in Figure 1. Additionally, zoom-in images stained with a polyclonal fibronectin antibody, the tensional probe (Cy5-FnBPA5), and DAPI are presented to visualize all fibronectin fibers, untensed fibronectin fibers, and nuclei, respectively.

The pink signal in the H&E-stained microthrombi reveals the presence of cytoplasmic proteins and ECM fibers (Figure 1A), along with darker regions containing a high number of cell nuclei. Cryosections of the microthrombi show an abundance of fibronectin fibers, with a high enhancement in untensed fibronectin (Figure 1A). While the Cy5-FnBPA5 signal (untensed fibronectin fibers) appears rather homogeneously distributed in the stitched IF overview image, a zoomed-in view reveals heterogeneous patches of fiber relaxation, with some areas highly stretched and others highly untensed (Figure 1B). This observation is further supported by the ratiometric image analysis of fibronectin and Cy5-FnBPA5 signals (Figure 1C). On average, 40% of all fibronectin fibers are untensed, as revealed by the ratiometric analysis of Cy5-FnBPA5^+^ pixels, normalized to the total number of positive pixels for the fibronectin polyclonal antibody (Figure 1D).

### Platelets are found in close contact to stretched fibronectin fibers, while further away from untensed fibers in the stroma of microthrombi

**Figure 2:**
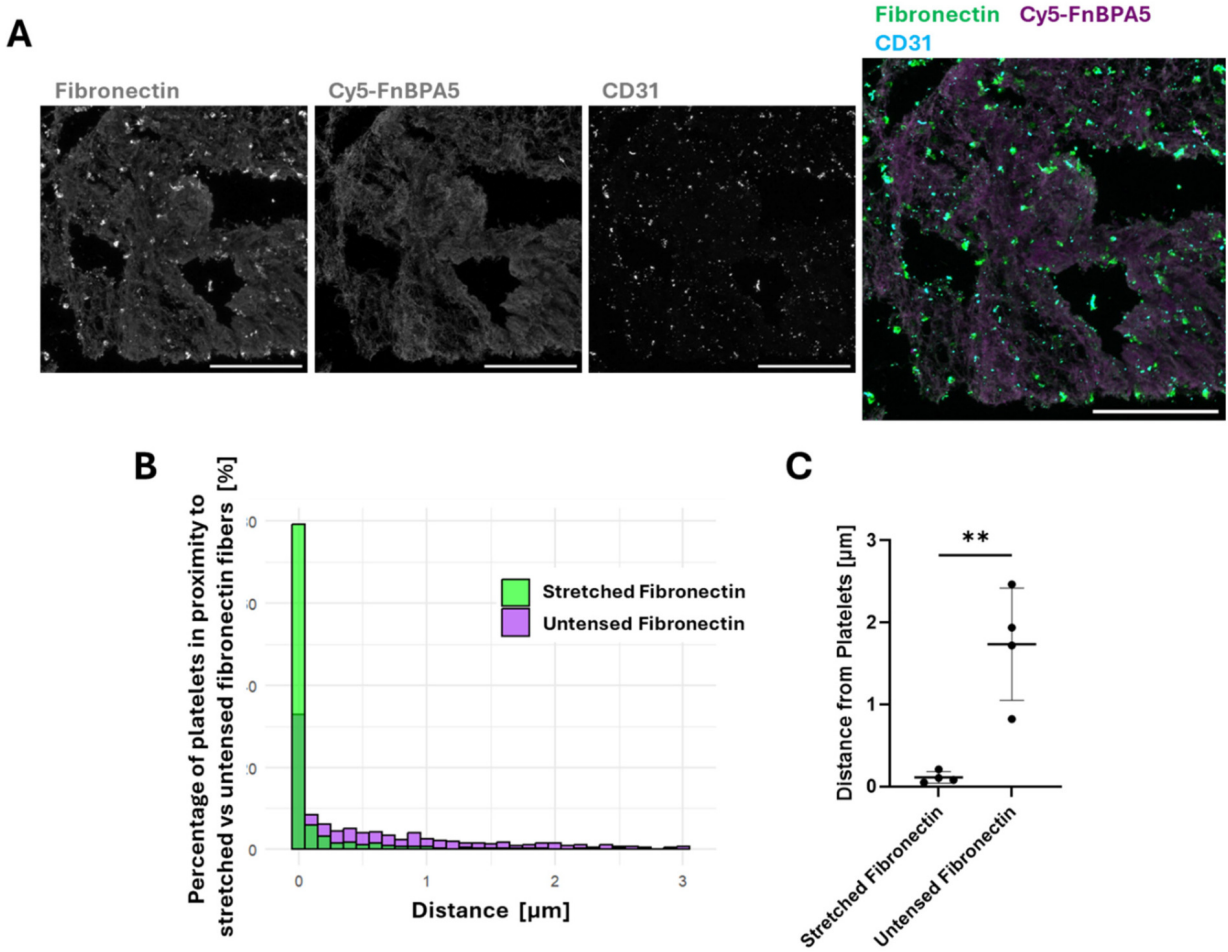
Platelets are found in closer proximity to highly stretched fibronectin fibers than to untensed ones in the stroma of microthrombi. **(A)** Representative confocal images stained with a polyclonal fibronectin antibody (green) to visualize the presence of all fibronectin fibers, co-stained with Cy5-FnBPA5 tension probe (magenta) to visualize the untensed fibronectin fiber pixels and CD31 to visualize the platelets. Scale bar: 100 µm. **(B)** Spatial proximity analysis of CD31^+^ platelets to stretched (green) and untensed (magenta) fibronectin fiber pixels showed the percentage of cells in close proximity to fibronectin fibers, depending on their tensional states. **(C)** Average distance of CD31^+^ platelets to fibronectin fibers of different strains: stretched or untensed. Each dot represents a single microthrombi, with multiple areas analyzed for each microthrombi. Mean ± SD. Unpaired Student's *t* test. *P*-value: ** < 0.01.

Observing fibronectin fiber relaxation was surprising, as surface-exposed platelets typically assemble highly stretched fibronectin nanofibrils upon activation (26). We thus asked where the platelets are located in these microthrombi in relationship to the stretched vs. untensed fibronectin fibers. IF was performed using CD31, also known as platelet endothelial cell adhesion molecule-1 (PECAM-1), a marker expressed by endothelial cells, as well as by platelets and various lymphocytes, including monocytes. While platelet activation drives thrombus formation, and their activation gets further enhanced by thrombin (27), blood-circulating lymphocytes get entrapped in thrombi as the fibrin network is formed (28, 29), and surface-exposure of CD31 triggers preferential platelet-monocyte aggregation (30, 31). As the distribution of cellular components in blood clots is highly heterogenous, CD31 staining provides information about the localization of these cells with respect to fibronectin fibers. CD31 staining was thus performed in combination with a polyclonal fibronectin antibody and Cy5-FnBPA5 to visualize all fibronectin fibers and untensed fiber pixels, respectively (Figure 2A). To distinguish between nucleated cells such as leukocytes and endothelial cells, and non-nucleated platelets, DAPI was used to differentiate them. Spatial proximity analyses were then performed between CD31^+^ cells lacking DAPI staining (platelets) and both stretched and relaxed fibronectin fibers (Figure 2B). This analysis computes the distance between the center of the cell determined with CD31 stains, and the closest Cy5-FnBPA5^−^ pixel (stretched fibronectin fiber) or Cy5-FnBPA5^+^ pixel (untensed fibronectin fiber). This analysis revealed that ∼80% of platelets in microthrombi were in contact with stretched fibronectin fibers, while only ∼33% were directly in contact with untensed fibronectin fibers (Figure 2B). The data for this analysis came from the same set of images, meaning that a single platelet can be in contact with both stretched and untensed fibronectin fibers. The average distance of platelets to stretched fibers is close to 0 µm, whereas they are significantly farther from untensed fibronectin fibers, with an average distance of 2 µm (Figure 2C). These findings indicate a strong correlation between the position of platelets and fibronectin fiber tension within the microthrombi stroma, as most platelets are found in direct contact with stretched fibers. This implies that platelets, and potentially other leukocytes, may contribute to the stretching of fibronectin fibers. Particularly platelets are known to assemble highly stretched fibers which then surround platelet aggregates (26). Contractile cell forces originating from platelets, and perhaps from other leukocytes could further contribute to the tensing of fibronectin fibers. Fibronectin fibers then appear significantly more relaxed with increasing distance from platelets, which might be due to various factors, including fibronectin fiber proteolysis, the reduced exposure to platelet traction forces, or due to other remodeling factors present in the thrombus.

### FnBPA5 can be used to detect microthrombi in epicardial arteries after the onset of MINOCA

**Figure 3:**
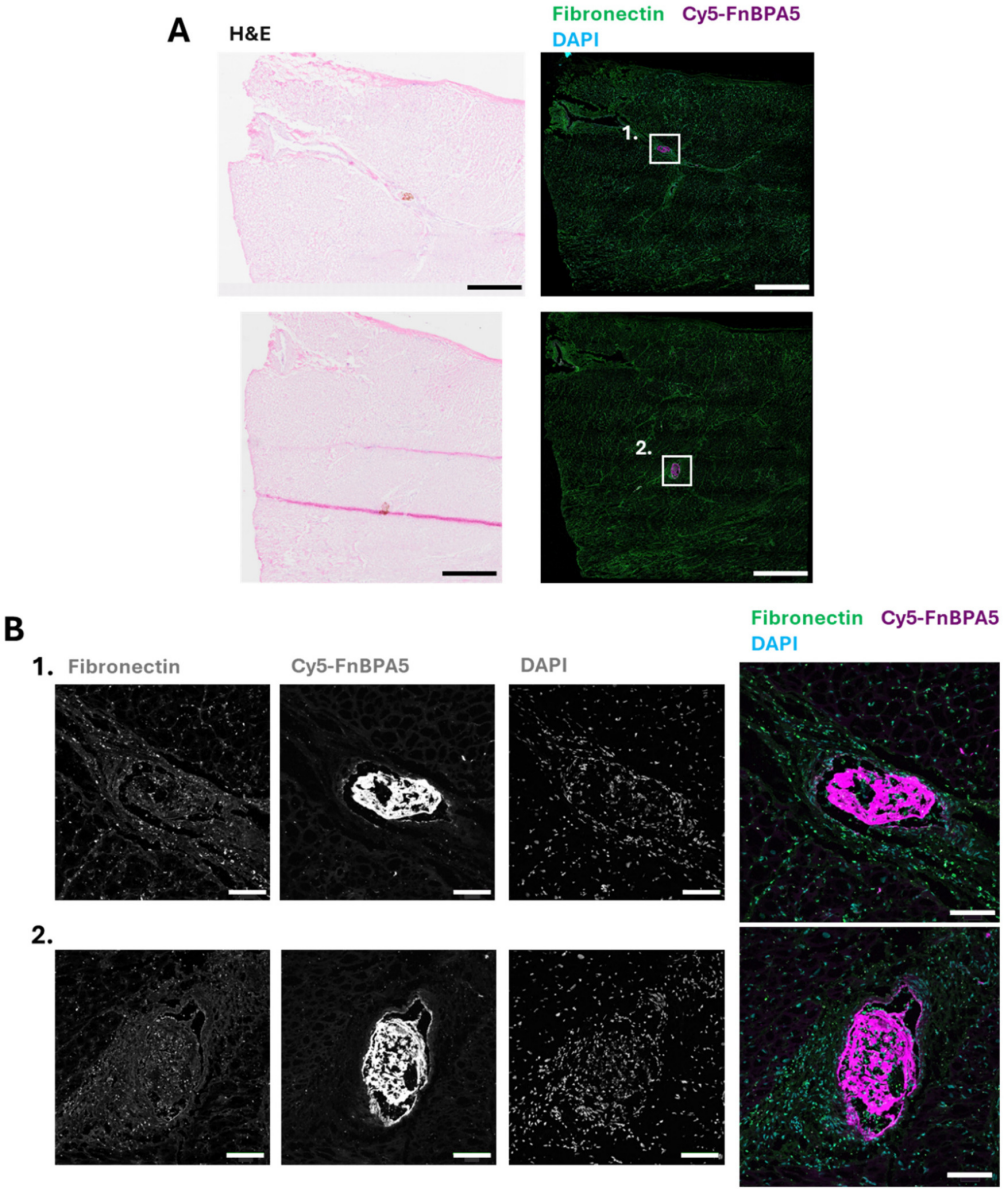
FnBPA5 allows to target the tensional signature of fibronectin fibers for the efficient identification of microthrombi in the cardiac muscle of a pig model. **(A)** Representative H&E staining image and confocal image of cryosections of porcine cardiac muscle with microthrombi, stained with a polyclonal fibronectin antibody (green) to visualize the presence of all fibronectin fibers, co-stained with Cy5-FnBPA5 tension probe (magenta) to visualize the untensed fibronectin fibers and DAPI to visualize the cells. Scale bar: 1,000 µm. **(B)** Higher resolution zoom-in confocal images (maximal intensity projection) stained with a polyclonal fibronectin antibody (green) to visualize the presence of all fibronectin fibers, co-stained with Cy5-FnBPA5 tension probe (magenta) to visualize the untensed fibronectin fibers and DAPI to visualize the cells. Scale bar zoom-in images: 100 µm.

As fibronectin is present in blood, blood clots, and fibronectin fibers are highly tensed in tissues from most healthy organs (13, 17), the presence of untensed fibronectin fibers in microthrombi, as seen in the section above, could enable precise identification of microthrombi by leveraging the contrast between untensed and stretched fibers. Therefore, as a next step, we stained for the presence of untensed fibronectin fibers in microthrombi after their injection into one of the main epicardial arteries thereby inducing the onset of MINOCA, as described by Cesarovic et al. (4). Ischemic cardiac tissues were harvested 6 h post microthrombi injection. Triphenyl tetrazolium chloride (TTC) staining was used to visualize infarcted areas in the whole heart, aiding in the localization of injected microthrombi. Infarcted regions were carefully collected, cryosectioned, and subjected to detailed H&E microscopic evaluation to precisely locate the injected microthrombi. After localization, IF was performed using a polyclonal fibronectin antibody co-stained with our tension probes (Cy5-FnBPA5) and DAPI to visualize untensed fibronectin fibers and nuclei, respectively (Figure 3). The high presence of untensed fibronectin fibers observed in microthrombi before injection (Figure 1) is also evident after injection into the coronary arteries, as shown in Figure 3A, as well as in the zoom-in images of microthrombi embedded in blood vessels (Figure 3B). Microthrombi distinctly stand out from the surrounding cardiac muscle, where fibronectin fibers get only stained by the fibronectin antibody confirming previous findings that fibronectin fibers are tensed in the healthy heart (13). This confirms the effectiveness of our tension probe to specifically target microthrombi. Previous studies have shown that fibronectin fibers are stretched not only in the heart but also in other healthy organ tissues (12, 13), while our results clearly demonstrate the presence of untensed fibronectin fibers within microthrombi. This contrast further validates the ability of our probe to differentiate microthrombi from myocardium based on fibronectin's fiber tension.

The ability to specifically target microthrombi in cardiac tissue after MINOCA onset using FnBPA5 represents a significant advancement for diagnosing microthrombi-induced MINOCA and offers potential therapeutic applications by enabling the targeted delivery of antithrombotic drugs directly to microthrombi.

## Discussion

MINOCA presents a significant diagnostic and therapeutic challenge due to its complex and heterogeneous nature (3, 4, 6). Conventional imaging and biomarker-based methods often fail to identify the underlying cause, highlighting the need for more refined diagnostic and therapeutic approaches to rapidly determine the etiology and improve patient management (4, 5).

To address these challenges, a pig model of autologous microthrombi-induced MINOCA was developed in 2023, providing a physiologically relevant platform for studying this specific form of MINOCA and for developing novel diagnostic and therapeutic approaches for better patient management (4). Using our peptide tension probe FnBPA5, whose nM affinity to fibronectin fibers is destroyed by fiber stretching (12, 13), we discovered here that fibronectin fibers are partially untensed in the stroma of microthrombi both before and after MINOCA onset, suggesting that fibronectin fiber relaxation may be a key marker of microthrombi and could potentially be used to improve patient management. Furthermore, we observed a strong correlation between the position of platelets and stretched fibronectin fibers, with platelets predominantly located near stretched rather than untensed fibronectin fibers in the stroma of microthrombi generated by carotid artery crush and collected after 60 min.

While it was initially surprising to observe the presence of untensed fibronectin fibers in circulating microthrombi, since platelets are key drivers of the assembly of highly stretched fibronectin fibers, which play a critical role in clot stabilization and tissue repair (10), this observation may be explained by platelet-driven remodeling processes. Activated platelets secrete matrix metalloproteinases (MMPs), such as MMP-2 and MMP-9, both of which can contribute to fibronectin fragmentation (32) and potentially induce fibronectin fiber relaxation. Leukocytes are also known to be present in the stroma of blood clots and to play a significant role in thrombus formation and maturation (33). Neutrophile, and to a lesser extent monocytes, also express MMPs as well as serine proteases such as cathepsin G, which is known to degrade fibronectin, beyond its other functions in blood coagulation (33, 34). A further explanation could thus be that the activated platelets within the thrombi progressively recruit circulating neutrophils (33, 35), which in turn triggers the release of further proteolytic enzymes that degrade ECM fibers, including serine proteases like neutrophil elastase (NE), proteinase 3 and cathepsin G, all of which are known to degrade fibronectin (36).

While this study highlights, for the first time, the significant presence of untensed fibronectin fibers in the stroma of circulating microthrombi, it has several limitations. First, it focuses exclusively on fresh microthrombi generated in a pig model. Second, we examined only acute MINOCA with a short induction period (6 h), thereby overlooking the effects of ischemia on surrounding tissues and blood clot maturation. Future studies should therefore investigate the tension state of fibronectin fibers in more mature thrombi, formed over longer time periods or even in human thrombi, as well as in ischemic tissues. This would be highly relevant for patient management, as it could help define the therapeutic window for the application of our peptide probes following partial vascular occlusion. A detailed investigation into the exact composition of microthrombi and the specific roles of various cell types in the relaxation of fibronectin fibers would provide valuable insights into the mechanisms underlying thrombus maturation and remodeling.

## Conclusion

Our discovery shows for the first time how the tensional signature of omnipresent proteins can be utilized for specific targeting in microthrombi. Our findings presented here open new avenues for using peptides that specifically bind to untensed fibronectin fibers as mechanical biomarkers of microthrombi in coronary arteries. The ability of FnBPA5 to specifically detect untensed fibronectin fibers makes it a promising tool for both diagnostic and therapeutic vascular applications. Like other peptides, FnBPA5 can be conjugated with contrast agents or radioligand for advanced imaging techniques, such as MRI or SPECT/CT, as demonstrated in previous studies (12, 18), to enhance microthrombus detection in patients. Additionally, it can be linked to thrombolytic agents for targeted drug delivery, offering a novel therapeutic strategy for dissolving microthrombi in affected blood vessels.

## Materials and methods

### Sample collection and processing

Animal experiments were performed on female domestic pigs (82 ± 5 kg; 4–5 months old) by veterinarians from the Center for Preclinical Development, University Hospital Zurich, and the Department of Health Sciences and Technology, ETH Zurich, Zurich, Switzerland. Animal studies were approved by the Veterinary Office (License ZH213/2019). The development and validation of the translational autologous microthrombi-induced MINOCA pig model are fully described in Cesarovic et al. (4). Briefly, microthrombi were produced by multiple carotid artery crushes and collected after 60 min. Some microthrombi were directly flash frozen in liquid nitrogen and stored at −80°C for cryosectioning and IF staining. After filtration, microthrombi (<200 µm) were injected into one of the main epicardial arteries, and the pig was monitored for 5 h. Ischemic tissues were stained with Triphenyl tetrazolium chloride (TTC) to facilitate the identification of injected microthrombi. After 6 h, the animal was euthanized, and the heart was harvested for histopathological analysis. Ischemic areas, as seen with TTC staining, were harvested, flash frozen in liquid nitrogen and stored at −80°C until cryosectioning.

### Hematoxylin and eosin (H&E) staining

H&E staining was performed on microthrombi and ischemic cardiac tissue sections of 10 µm thickness using the linear stainer COT 20 (Medite, Germany), which performs automated H&E staining. After staining, slides were mounted using Eukitt (Sigma-Aldrich) and imaged using the Olympus VS200 slide scanner.

### Immunofluorescence (IF)

Microthrombi and ischemic cardiac tissue were sectioned at 10 µm using a cryostat and placed directly onto microscope slides. Slides were stored at −80°C until staining. Cryosections were stained for specific ECM markers and for the Cy5-FnBPA5 tension probe following the previously developed protocol (12, 13, 16). Briefly, cryosections were first blocked 30 min with 4% bovine serum albumin (BSA) before being incubated for 1 h at room temperature with 5 µg/ml of Cy5-FnBPA5 or Cy5-labeled scrambled-FnBPA5 as control. Sections were further washed and fixed with 4% paraformaldehyde (PFA) in 1xPBS for 10 min. Tissue sections were further blocked with 5% goat serum with 0.3 M glycine for 1 h and later incubated with primary antibodies overnight at 4°C. Secondary antibodies were then applied for 1 h at room temperature and some sections were further stained with DAPI 2 µg/ml for 10 min before being mounted with a hardening DAKO Fluorescence mounting medium (DAKO, Denmark). The stained and mounted slides were imaged after 24 h using a confocal microscope (Leica SP8).

### Confocal imaging of IF-stained microthrombi and tissue sections

Stained cryosections were imaged with a Leica SP8 confocal microscope. Full tissue cryosections overviews were acquired with a 10x objective and further zoom-in of specific areas was done with higher resolution, using a 20x objective.

### Ratiometric image analysis to visualize untensed vs. tensed fibronectin fibers

Ratiometric image analysis of Cy5-FnBPA5 staining relative to total fibronectin was performed using a custom program written in MATLAB (Natick, MA, USA). Briefly, images were thresholded using Otsu's method, and masked based on where both fibronectin and Cy5-FnBPA5 signals passed the threshold. The fluorescence intensities of Cy5-FnBPA5 vs. total fibronectin were calculated.

### Proximity analyses between platelets and fibronectin fibers (Cy5-FnBPA5^+^; Cy5-FnBPA5^−^)

Confocal images of zoom-in areas of cryosections stained with the Cy5-FnBPA5, a fibronectin polyclonal antibody, and the platelets marker CD31 were processed in Qupath (37). The detection of untensed/relaxed fibronectin fibers was based on a machine learning approach. To train the model, several training images were randomly selected on zoom-in areas of cryosections stained with Cy5-FnBPA5. For each class, negative (non-untensed fibronectin fibers) and positive (untensed fibronectin fibers), manual annotations were performed until the model was successfully able to recognize relaxed fibronectin fibers. After successful training of the model, the classifier was then applied to tissue sections, and masks with positive pixels for untensed and stretched fibronectin fibers were created. Platelets were detected using the built-in function “cell detection” and identified based on their CD31 signal. Since CD31 is not specific to platelets alone, DAPI was used to distinguish nucleated cells, such as leukocytes, from platelets. Finally, the proximity analysis between CD31^+^ cells and both stretched and untensed fibronectin fibers was performed using Qupath's built-in function “spatial analysis- distance to annotations 2D”, which calculates the smaller distance between cells and Cy5-FnBPA5 positive or negative pixels. This analysis computes the distance between the center of the cell, and the closest positive/negative pixel of Cy5-FnBPA5.

### Statistical analysis

Statistical analyses were performed using GraphPad Prism 10.1.2. Parametric or non-parametric distribution was assessed based on four normality tests, D'Agostino-Pearson, Anderson–Darling, Shapiro–Wilk and Kolmogorov–Smirnov, and based on QQplot graphical assessment as provided by GraphPad Prism. Statistical significance of two parametric groups was performed using the unpaired Student's *t* test, while two non-parametric groups were analyzed using the Mann–Whitney test.

## Data Availability

The raw data supporting the conclusions of this article will be made available by the authors, without undue reservation.
